# Retrieval of entrapped Rotablator burr in coronary stent using guide extension catheter and RotaWire spring-tip

**DOI:** 10.1007/s12928-023-00939-6

**Published:** 2023-05-30

**Authors:** Takayoshi Toba, Tomoyo Hamana, Hiroyuki Kawamori, Sho Torii, Gaku Nakazawa, Hiromasa Otake

**Affiliations:** 1 Division of Cardiovascular Medicine, Department of Internal Medicine, Kobe University Graduate School of Medicine, 7-5-2, Kusunoki-cho, Chuo-ku, Kobe, 6500017 Japan; 2 Department of Cardiology, Tokai University School of Medicine, Isehara, Japan; 3 Division of Cardiology, Department of Internal Medicine, Kinki University School of Medicine, Osaka, Japan

## Case presentation

**Fig. 1:**
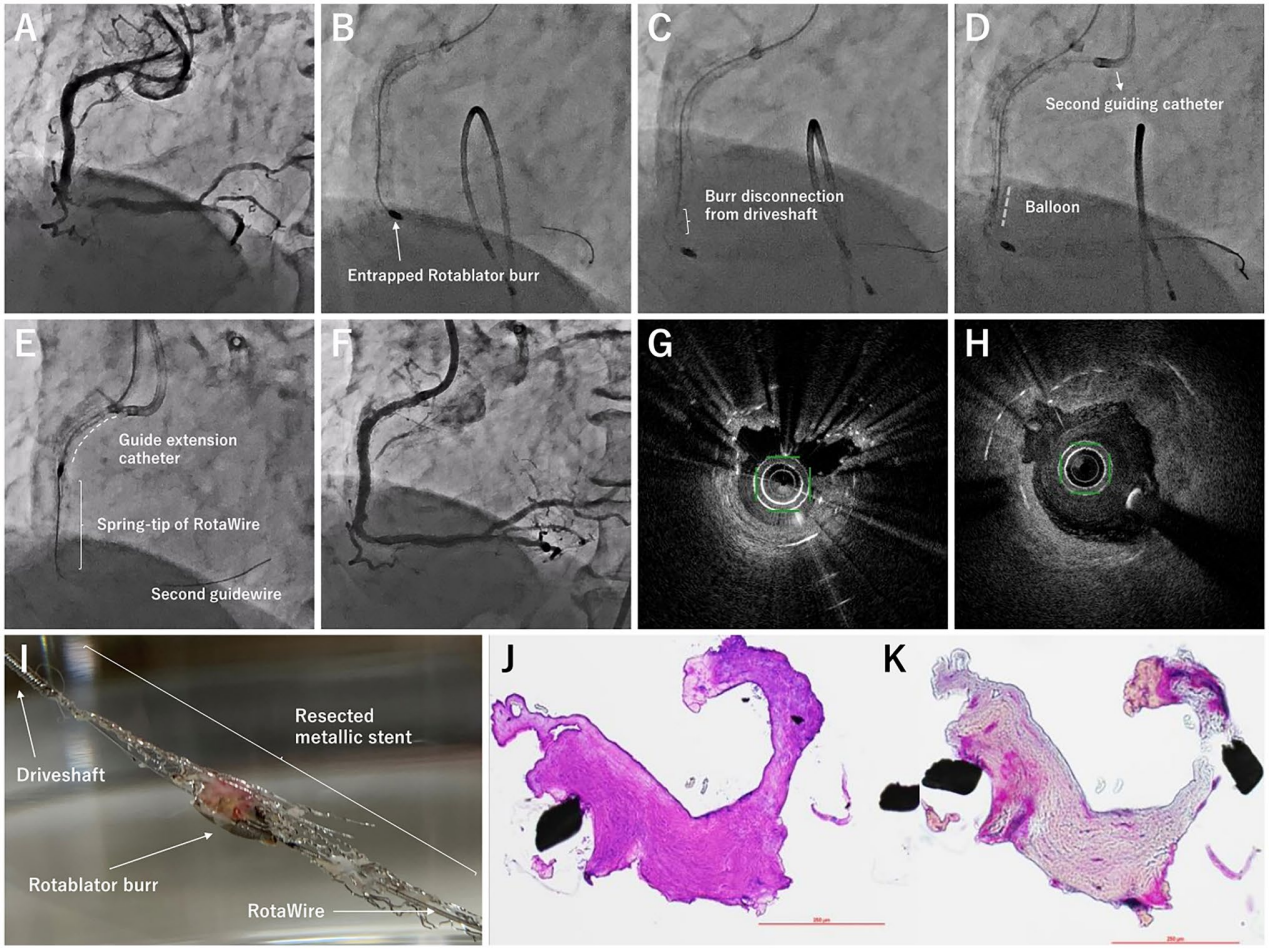
HYPERLINK "sps:id::fig1||locator::gr1||MediaObject::0" **A**–**F** Coronary angiographic findings. **A** Initial, **B** Burr entrapment, **C** Burr disconnection from driveshaft, **D** Balloon dilatation beside the entrapped burr, **E** Retrieval of the entrapped burr, **F** Final. **G**, **H** Optical coherence tomography findings before (**G**) and after percutaneous coronary intervention (**H**) **I** Macroscopic findings of the retrieved Rotablator burr. **J**, **K** Microscopic findings of the neointima attached to the stent strut. **J** Hematoxylin and eosin staining, **K** Movat Pentachrome staining

A 78-year-old man underwent coronary angiography due to medically refractory effort angina. He previously underwent multiple percutaneous coronary interventions (PCIs), without debulking devices, for in-stent restenosis (ISR) of the right coronary artery (RCA). Angiography revealed ISR in the middle RCA (Fig. 1A). Optical coherence tomography (OCT) showed stent underexpansion with stent fracture, which mainly caused ISR (Fig. 1G). Rotational atherectomy (RA) was performed with 7-French guiding catheter and RotaWire Extra Support (Boston Scientific Corporation, Natick, MA, USA) via femoral approach. When the 1.75-mm Rotablator burr (Boston Scientific Corporation) was advanced toward the proximal portion of the lesion, the burr was entrapped, followed by its disconnection from the driveshaft (Fig. 1B, C, Supplementary Movie 1). Using the double-guiding catheter technique, balloon on another guidewire crossing via the second guiding catheter was dilated beside the entrapped burr (Fig. 1D). Finally, RotaWire, the distal enlarged spring-tip of which was united by the burr, was pulled as the guide extension catheter over the driveshaft was pushed forward, and then the burr was successfully retrieved (Fig. 1E*,* Supplementary Movie 2). Final OCT findings demonstrated that the stent struts covered with neointima had resected (Fig. 1H). The target lesion was treated with drug-coated balloon without any complications (Fig. 1F). Macroscopic examination of the retrieved burr showed that the metallic stent, to which neointimal tissue was attached, was clinging to the driveshaft and the disconnected burr (Fig. 1I). Pathological examination revealed collagen-rich neointimal tissue without calcified or lipid components (Fig. 1J, K).

RA is a feasible treatment option for ISR lesions due to stent underexpansion and fracture [1]. However, our case implies that indication of RA should be decided carefully, especially in those with severe tortuosity. Depending on cases, we should prioritize medical titration and avoid debulking device including RA. In the present case, we decided to use RA because of medically refractory symptom and a history of repeated PCI without debulking devices. The macroscopic findings showed that the burr entangled in stent struts is the main mechanism of burr entrapment in ISR. The RotaWire was pulled tightly, generating the strong pulling force of the Rotablator burr entangled in stent struts. Moreover, pushing force with guide extension catheter supported to remove the entrapped burr successfully. This technique might be a reasonable strategy to retrieve the entrapped burr in ISR lesions, although we should consider the potential risk of coronary rupture. PCI operators using RA should be aware of this bailout technique.

## Supplementary Information

Below is the link to the electronic supplementary material.

Supplementary file1 (MP4 3185 KB)

Supplementary file2 (MP4 4471 KB)

## Data Availability

Data sharing is not applicable to this article as no new data were analyzed in this study.
